# Health-promoting lifestyles of university students in Mainland China

**DOI:** 10.1186/1471-2458-9-379

**Published:** 2009-10-09

**Authors:** Dong Wang, Chun-Quan Ou, Mei-Yen Chen, Ni Duan

**Affiliations:** 1 Department of Health Service Management, School of Public Health and Tropical Medicine, Southern Medical University, Guangzhou, China; 2 Department of Biostatistics, School of Public Health and Tropical Medicine, Southern Medical University, Guangzhou, China; 3 Department of Nursing, Chang Gung Institute of Technology, Taipei, Taiwan; 4 Center of Network & Information, Southern Medical University, Guangzhou, China

## Abstract

**Background:**

Health-promoting lifestyles of adolescents are closely related to their current and subsequent health status. However, few studies in mainland China have examined health-promoting behaviors among university students, notwithstanding the dramatic development of higher education over the past two decades. Moreover, no study has applied a standardized scale to such an investigation. The adolescent health promotion (AHP) scale has been developed and is commonly used for measuring adolescent health-promoting lifestyles in Taiwan. The aim of this study is to determine the appropriateness of the AHP for use in mainland China.

**Methods:**

A cross-sectional study was performed on a total of 420 undergraduates, who were randomly selected using a two-stage stratified sampling method in a university in Guangzhou city, mainland China. The simplified Chinese version of the AHP scale, comprising six dimensions (Nutrition behavior, Social support, Life-appreciation, Exercise behavior, Health-responsibility and Stress-management), was used to measure health-promoting lifestyles among undergraduates. The reliability of the AHP scale was assessed using split-half reliability coefficients, intraclass correlation coefficients and Cronbach's α coefficient. Validity was assessed by factor analysis and correlation analysis. Factors associated with health-promoting lifestyles were identified using multiple linear regression.

**Results:**

Cronbach's coefficients were greater than 0.7 in all dimensions of the AHP scale except for Nutrition behavior (0.684). Intraclass correlation coefficients ranged from 0.689 to 0.921. Split-half reliability coefficients were higher than 0.7 in three AHP dimensions (Social support, Life-appreciation and Exercise behavior). Our results were generally in accordance with the theoretical construction of the AHP scale. The mean score for each of the six dimensions was lower than 70. Gender and grade were the factors primarily associated with health-promoting lifestyles among undergraduates.

**Conclusion:**

The AHP is a valid and reliable instrument for assessing health-promoting lifestyles of undergraduates in mainland China, which remain at a low level. Health behavior education taking account of gender and grade differences may also be applied.

## Background

The World Health Organization points out that 60% of the quality of an individual's health and life depends on his/her behavior and lifestyle [1]. Health-risk behaviors are activities that increase a person's vulnerability or susceptibility to negative health outcomes [2]. In contrast, health promoting behaviors entail a positive approach to living and a means of increasing well-being and self-actualization [3]. Numerous publications have shown that practicing health promotion behaviors decreases the occurrence of disease and lowers the death rate [4,5]. A good health-promoting behavior depends on the living habits adopted during early years. Adolescents are at a dynamic transition period bridging childhood to adulthood, characterized by rapid, interrelated changes in body, mind and social relationships [6]. There is a great deal of evidence that adolescents, particularly those in the age range 15-24, engage in health-risk behaviors such as smoking, drinking, having unprotected sexual intercourse, carrying weapons and adopting poor eating habits. These behaviors lead to a variety of adverse health outcomes, including major morbidities and mortalities among that age group [7,8], and will be carried into adulthood, jeopardizing their health status in later life [9]. Many effects of health-risk factors among adults are avoidable if these behaviors are identified and changed at an early stage [10,11]. Therefore, it is essential to understand and evaluate health-promoting behaviors among adolescents in order to promote their healthy growth. In particular, this is an ideal time to conduct health education for adolescents in a higher education environment in terms of cost-effectiveness.

Many studies have shown that several factors at the individual, family and community levels influence an adolescent's risk for engaging in health-risk behaviors [12-14]. However, there are fewer studies of adolescent health-promoting behaviors and particularly less information about the associated factors. A few studies have consistently shown a gender difference in health-promoting behaviors [15]. Other factors that may be relevant include age, ethics, examination level at admission and educational level of their parents [16-18].

To date, a few instruments have been developed to evaluate health-promoting lifestyles: Health-Promoting Lifestyle (HPLP) [19], Adolescent Lifestyle Questionnaire (ALQ) [20], Adolescent Lifestyle Profile (ALP) [21] and Adolescent Health-Promoting (AHP) scale [22]. The AHP scale based on the Pender's Health Promotion Model has been developed by Chen for evaluating adolescent health-promoting lifestyles. It has been widely used in Taiwan as a useful tool for investigating the health promoting behaviors of adolescents and evaluating the effects of health education [23-29]. It has also been translated into many languages and its content has been examined cross-culturally [30].

The development of higher education in mainland China has been very dramatic in recent years. For example, there were 5.56 million academy students and undergraduates in 2000. The number tripled (to 15.62 million) in 2005 and is expected to increase up to 20 million by 2010 [31]. More and more students tend to practice health-risk behaviors such as smoking, alcohol drinking, sedentary lifestyle and irregular breakfasting [32-35]. However, there have been few studies on health-promoting lifestyles among university students in mainland China. Moreover, no study so far has proposed or applied a standardized scale for examining such behaviors in this population.

Our aim in the present study is to examine the reliability and validity of the AHP scale for application to undergraduates in mainland China, investigating the prevalence of health-promoting behaviors and identifying the associated factors.

## Methods

### Subjects

The subjects were chosen from a total of 4523 undergraduates in Southern Medical University, located in Guangzhou, China. The university offers 22 specialties, stratified into nine medicine specialties and 13 non-medicine specialties. In this study, a two-stage stratified sampling method was applied. First, we applied a proportionate allocation strategy to sample two medicine specialties and three non-medical specialties, which were selected randomly on the basis of the discipline category-specialty sequence. Next, all the student numbers in the selected specialties were listed. A random sample of 100 student numbers was drawn from each selected specialty; 421 out of the total of 500 selected subjects agreed to be recruited for this investigation. The overall response rate was 84.2%. To evaluate the reliability of the results, 10% of the total respondents (44) were randomly selected for a retest 7 days after the baseline test.

### Questionnaire

In view of the similarity in social and cultural, ethnic and demographic characteristics between Taiwan and mainland China, we adopted the AHP scale to assess the health-promoting lifestyles of university students. A detailed description of this scale can be found in previous publications [22]. Briefly, it comprises 40 items assessing six dimensions of behavior: (1) nutrition; (2) social support; (3) life-appreciation; (4) health responsibility; (5) stress-management; and (6) exercise behavior. The frequency of reported behaviors was obtained using a self-reporting Likert scale with a five-point response format, "never, rarely, sometimes, usually, always", with the rating score ranging from 1 to 5.

In this study, the AHP scale was converted to a simplified Chinese version, then two linguists and Mei-Yen Chen, the developer of the scale, verified the accuracy of translation. As the AHP had not been used previously in mainland China, a pilot test was performed to determine whether it would be appropriate for our students. Thirty students completed the simplified Chinese AHP questionnaire for the purpose of testing the clarity and relevance of the statements. All items were understood perfectly and were completed without difficulty.

In addition, we collected some demographic information about each respondent: gender, grade, specialty, family residence, nationality, and monthly family income. The grade was divided into two categories: senior students (who had been in the university for 3~5 years) and junior students (who had been in the university for only 1~2 years). The family residence was classified into rural and urban areas on the basis of the local citizenship residence registered before enrollment.

### Field work

Ethical approval was obtained prior to conducting this study. All respondents signed a written informed consent before participation, and each respondent was free to discontinue participation at any time. Because the study was intended to identify the usual pattern of university students' health practices and to avoid the confounding effects of seasonal holidays and the stressful period of examination time, the survey was conducted from November to December 2007, in the middle of the semester, via a self-administered questionnaire. To maximize the response rate without allowing the researchers to influence the respondents, all questionnaires were delivered and collected face-to-face by students trained as interviewers by the researchers. The respondents filled in the questionnaires by themselves. The interviewers on the site explained any unclear questions without inducement, if necessary. To examine the test-retest reliability of the questionnaire, about ten percent (44) of the respondents completed the questionnaires again a week later.

### Data management

All valid questionnaires were input doubly into the database by two independent postgraduates using EpiData 3.1 software. The data were cleaned after double entry. We conducted computer logical checking and also manual checking, particularly when there were discrepancies between the two operators. In total, 14 invalid questionnaires were discarded because of incompleteness or double answers. Among these, nine had incomplete responses (i.e. more than 80% of the items were missing) and five had double answers to an item. Finally, a total of 407 valid questionnaires (96.9% of the total) were used for data analyses in this study. Of the 44 respondents who agreed to be re-interviewed, three questionnaires were rejected because they were not completed in line with the study protocol, resulting in 41 questionnaires for the retest analysis.

The missing values were handled using the method described in previous studies [36]. In our survey, the item response rates were actually quite high. The average item response rates were 93.21% for the general information and 99.37% for the 40 items in AHP (range 98.45-99.91%). The raw score for each of the six AHP dimensions was derived by summing the item scores, and converted to a value for the dimension from 0 to 100. It was then re-calculated across the dimension as follows:



The AHP questionnaire was evaluated for reliability and validity. Split-half reliability was computed by correlating the scores of the odd half with those of the even half in each dimension of AHP. Test-retest reliability was assessed by the differences between test and retest scores using a paired-sample t test and was further assessed by the intraclass correlation coefficient (ICC). The internal consistency of the AHP items was assessed by Cronbach's *α *coefficient. Construct validity was assessed by correlation analysis and factor analysis using principal component analysis [37]. Student's t test and multiple linear regressions were applied to investigate the effects of individual characteristics on the health-promoting lifestyles of undergraduates. All these statistical methods were implemented via SPSS 17.0. The figures thus obtained were plotted using R project.

## Results

### Characteristics of subjects

The ages of the subjects in this study ranged from 16 to 25 years with a mean age of 21 years. Of the 407 students who completed the questionnaires, 183 (44.96%) were males and 224 (55.04%) females. In more detail, there were 200 (49.14%) senior students and 207 (50.86%) junior students; 223(54.79%) came from rural areas and 184(45.21%) from urban areas; 271(66.58%) were majoring in a medicine discipline and 136 (33.42%) in a non-medicine discipline.

### Reliability

#### 1. Split-half reliability test

Three of the six AHP dimensions (Social support, Life-appreciation and Exercise behavior) had split-half reliability coefficients higher than 0.7, while the other three dimensions (Nutrition behavior, Health-responsibility and Stress-management) had coefficients ranging from 0.6 to 0.7. The lowest split-half reliability coefficient (0.61) was observed for the dimension of Nutrition behavior (Table 1).

**Table 1:** Reliability and correlation of the AHP dimensions

		**Reliability**				**Correlation**	
		
**Dimension**	**Item amount**	**Split-half reliability**	**Test-retest mean difference**	**ICC**	**Cronbach's α**	**Correlations between dimensions and items inside**	**Correlations between dimensions and items outside**
Nutrition behavior	6	0.610	-0.459	0.689	0.684	0.540-0.655	0.071-0.366
Social support	7	0.716	-0.237	0.809	0.828	0.672-0.725	0.091-0.510
Health-responsibility	8	0.672	-2.491	0.878	0.768	0.518-0.690	0.054-0.406
Life-appreciation	8	0.830	1.125	0.902	0.880	0.678-0.786	0.124-0.546
Exercise behavior	5	0.764	-0.558	0.882	0.834	0.588-0.866	0.008-0.367
Stress-management	6	0.697	2.146	0.921	0.757	0.615-0.709	0.089-0.465

#### 2. Test-retest reliability

The absolute mean differences between the test and retest scores ranged from 0.237 to 2.491. The paired-sample t test indicated that the difference was not statistically significant for any of the six dimensions *(P > 0.05)*. The one-week ICC ranged from 0.809 (the Social support dimension) to 0.921 (the Life-appreciation dimension) for five of the six dimensions; the exception was the Nutrition behavior dimension, which had a one-week ICC of 0.689 (Table 1).

#### 3. Cronbach's α

The internal reliability of the AHP scale was measured by Cronbach's *α *coefficient. The instrument showed high internal consistency (*α *= 0.92) overall (Table 1). Five of the six AHP dimensions (Social support, Life-appreciation, Exercise behavior, Health-responsibility, and Stress-management) had *α *coefficients higher than 0.7, the exception being Nutrition behavior (*α *= 0.684) (Table 1).

### Validity

#### 1. Factor analysis

Construct validity was demonstrated for the AHP questionnaires using factor analysis, a common approach to exploring whether the predicted factor structure of a questionnaire is supported. Before the exploratory analysis, both the Kaisor-Meyer-Olkin (KMO) and Bartlett's sphericity tests were used to measure the adequacy of sampling. The results showed that the KMO value was 0.89, and the significance of Bartlett's sphericity was 0.000 (*χ*^2 ^= 6650, *p *< 0.001), indicating that the samples met the criteria for factor analysis [38]. Principal component analysis was performed using Varimax rotation with Kaiser normalization. Factor analysis yielded a 6-factor solution with an explained variance of 61.92%, with eigenvalues greater than 1.00. Factor analysis of the 40-item questionnaire indicated that every item had a load value higher than 0.4 corresponding to factor and communality, as shown in Table 2. Seven of the 40 items (17.5%) had communalities lower than 0.5, while the other 33 had communalities in the range 0.504-0.791, indicating that most items were explained by their respective common factor. All items had load values ranging from 0.442 to 0.879, which are higher than the minimum criterion (0.4) of the construct validity test [37].

**Table 2:** Factor Loadings and Factor Structure of AHP

**Dimension/Item**	**F1**	**F2**	**F3**	**F4**	**F5**	**F6**	**communality**
**Nutrition behavior**							
1.Eat three regular meals							0.615
2.Make an effort to select foods without too much oil						0.719	0.626
3.Include dietary fiber						0.706	0.609
4.Drink at least 1,500 cc of water daily						0.591	0.566
5.Include five food groups in each meal						0.449	0.430
6.Eat breakfast daily							0.700
**Social support**							
7.Express my caring and warmth to others				0.764			0.695
8.Concern about and keep in touch with others				0.840			0.772
9.Discuss my concerns with others				0.680			0.569
10.Make an effort to smile or laugh every day				0.564			0.630
11.Enjoy keeping in touch with relatives				0.674			0.672
12.Maintain good interpersonal relationship				0.447			0.567
13.Talk about my troubles with others				0.462			0.504
**Health-responsibility**							
14.Read food labels at every purchase			0.538				0.485
15.Make an effort to moderate my body weight			0.673				0.537
16.Discuss my health concerns with health personnel			0.571				0.464
17.Observe my body at least monthly			0.667				0.550
18.Brush my teeth and use dental floss after meals			0.634				0.418
19.Wash hands before meals							0.485
20.Search for health information			0.508				0.582
21.Make an effort to choose foods without additives							0.552
**Life-appreciation**							
22.Make an effort to like myself	0.601						0.507
23.Make an effort to feel happy and content	0.713						0.674
24.Make an effort to feel growth in a positive direction	0.733						0.667
25.Make an effort to understand my strengths, weaknesses and accept them	0.634						0.580
26.Make an effort to audit my own defects and correct often	0.609						0.527
27.Make an effort to know what's important for me	0.577						0.489
28.Make an effort to feel interesting and challenge every day	0.764						0.656
29.Make an effort to believe that my life has purpose	0.754						0.676
**Exercise behavior**							
30.Perform stretching exercise daily		0.741					0.670
31.Exercise rigorously 30 min at least 3 times per week		0.879					0.791
32.Participate in physical fitness class at school weekly		0.812					0.705
33.Warm up before rigorous exercise		0.718					0.611
34.Make an effort to stand or sit straight	0.418	0.442					0.452
**Stress-management**							
35.Make an effort to spend time daily for muscle relaxation					0.657		0.617
36.Make an effort to determine the source of each stress that occurs					0.741		0.689
37.Make an effort to monitor my emotional changes					0.623		0.592
38.Sleep 6-8 hr each night					0.763		0.684
39.Make schedules and set priorities					0.707		0.662
40.Use adequate responses to unreasonable issues					0.456		0.558

#### 2. Correlation analysis

As shown by Spearman correlation analysis, items within a single dimension correlated more highly with the total score of the dimension (to which they were conceptually related) than with the dimensions to which they were conceptually unrelated (Table 1).

### Health-promoting lifestyles of undergraduates

Table 3 shows the values of the AHP dimension scores. The mean value of the total score of health-promoting lifestyles among undergraduates was 62.84, and the average scores of all AHP dimensions were lower than 70. The highest mean value (69.97) was observed for Life-appreciation, followed by Social support, Stress-management and Nutrition behavior, which had very similar mean values. The mean scores of the Health-responsibility and Exercise behavior dimensions were lower than 60.

**Table 3:** Descriptive summary of AHP scores in each dimension

**Dimension**	**Mean**	**SD**	**Rating**
Nutrition behavior	66.46	16.02	4
Social support	68.29	16.45	2
Health-responsibility	55.61	17.71	5
Life-appreciation	69.97	17.09	1
Exercise behavior	45.29	24.20	6
Stress-management	67.59	16.29	3

### Association with individual characteristics

**Figure 1:**
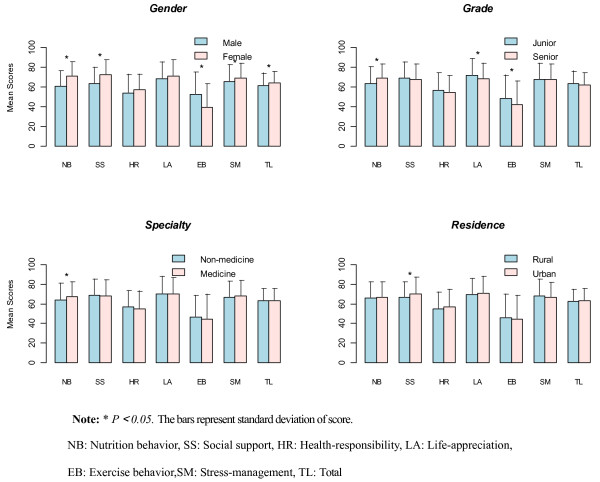
**Mean scores of each dimension by individual characteristics**. *P < 0.05. The bars represent standard deviation of score. NB: Nutrition behavior, SS: Social support, HR: Health-responsibility, LA: Life-appreciation, EB: Exercise behavior, SM: Stress-management, TL: Total

The two-sample t-test revealed a statistically significant difference in at least one dimension of AHP by gender, grade, specialty or family residence (Figure 1). Overall, the health-promoting lifestyles of male undergraduates were poorer than those of female undergraduates, and the difference was statistically significant (*P *< 0.05). Female undergraduates scored slightly higher on Nutrition behavior, Social support and Stress-management than males, and the difference was statistically significant (*P *< 0.05). Female undergraduates also scored slightly higher than male undergraduates on Life-appreciation and Health-responsibility, but these differences were not significant (*P *> 0.05). In contrast, male undergraduates were more actively engaged in physical activities than female undergraduates (*P *< 0.01). Junior undergraduates scored lower than senior students on the Nutrition behavior dimension, and the difference was statistically significant *(P = 0.01)*, while junior undergraduates had better health-promoting lifestyles in Life-appreciation and Exercise behavior dimensions than senior undergraduates (*P < 0.05)*.

Table 4 presents the multiple linear regression results using full and stepwise models. Both types of model showed that no AHP dimension was statistically associated with specialty and family residence (Table 4). All dimensions except Health-responsibility and Life-appreciation were associated with gender in the full models. Gender was significant in the stepwise models except in the Health responsibility dimension. Grade was significantly associated with Nutrition behavior and Life-appreciation in both the full and stepwise models.

**Table 4:** Association between demographical characteristics and AHP scores

	**Gender**		**Grade**		**Specialty**		**Residence**	
				
	**full models**	**stepwise models**	**full models**	**stepwise models**	**full models**	**stepwise models**	**full models**	**stepwise models**
Nutrition behavior	10.171**	10.124**	4.247*	4.224**	-0.071	-	-0.417	-
Social support	8.837**	8.618**	-1.927	-	-1.049	-	2.516	-
Health-responsibility	3.477	-	-2.301	-	-1.821	-	1.644	-
Life-appreciation	3.167	3.430*	-4.964**	-4.286*	1.767	-	1.145	-
Exercise behavior	-12.574**	-13.116**	-4.959	-	1.959	-	-0.413	-
Stress-management	3.634*	3.536*	-0.825	-	1.368	-	-1.746	-
Total	3.374**	3.168*	-1.897	-	0.245	-	0.622	-

*Note: *Gender (0 = Male, 1 = Female); Grade (0 = Junior, 1 = Senior); Specialty (0 = Non-medicine, 1 = Medicine; Residence (0 = Rural, 1 = Urban)

** P < 0.01; *P < 0.05.

## Discussion

Health-promoting lifestyle among adolescents has become a research focus worldwide. Life in college is a transitional period, offering good opportunities for establishing health-promoting lifestyles. Most research on health-promoting behaviors has been undertaken in the US and European countries, where university students are little engaged in health-promoting behaviors, especially healthy diet and physical activity [39-41]. However, data on health-promoting lifestyles among university students in mainland China are limited. To our knowledge, this is the first (albeit preliminary) study to investigate health-promoting behaviors of university students in mainland China using a standardized scale. Chen et al. reported that the reliability and validity of the AHP scale was very satisfactory in studies of health-promoting behaviors in Taiwan [22]. The present study evaluated the appropriateness of this scale for use with undergraduates, providing important information for a further large-scale investigation.

A Cronbach's *α *value of 0.7 or higher is generally considered sufficient to demonstrate internal consistency [42]. The Taiwan studies reported that the internal consistency of the AHP scale was higher than 0.7 on all six dimensions [22]. Similarly, the present study found *α *values above 0.7 for all dimensions except for Nutrition behavior, indicating that the internal consistency is satisfactory when the AHP scale is used in mainland China. The Nutrition behavior dimension had a relatively low Cronbach's *α *coefficient (0.684), and the same applied to ICC and split-half reliability, indicating there might be some problems in the conceptualization of Nutrition behavior. It may be difficult for undergraduates to assess their daily intakes of various kinds of food.

Factor loadings larger than 0.4 are usually considered to support the factor construction of a particular dimension [42]. Judged by this criterion, our factor analysis results indicated that the AHP scale accorded generally with the theoretical construction. Correlation analysis indicated that each of the 40 items was highly correlated with the hypothesized dimension, while relatively low correlations were observed between the items and other dimensions. In addition, consistent with previous studies, we found a significant gender difference in health-promoting behaviors, suggesting that this scale had good construct validity since it distinguished different subgroups as expected. Therefore, we may conclude that AHP was acceptable and applicable for evaluating the health-promoting lifestyles of adolescents in mainland China.

The results of this study showed that the mean scores of all dimensions of AHP were lower than 70. In particular, the mean scores on the Health-responsibility and Exercise behavior dimensions were lower than 60. This finding is consistent with the results of previous investigations in Taiwan [43,44] and Hong Kong [15]. A sedentary lifestyle is a common and serious problem among university students. Compared to young adults in general, the pressure of work is so severe for university students that much of their time and energy is likely to be occupied with their studies. On the other hand, the popularization of computers and the Internet may provide more choices of entertainment and reduce interest in exercise. Lack of exercise facilities is also a major reason why university students do not participate actively in exercise.

We found that the health-responsibility score was the second lowest among the six dimensions. Undergraduates in mainland China usually reside on campus with schoolmates, and are less likely to pay attention to their own health than younger adolescents such as primary and high school students who live with parents and are frequently reminded about health. Moreover, university life adds more stress and requires more independent decision-making by young people. They are also challenged to attain the personal growth and perseverance necessary to cope with life stress and to establish healthy interpersonal relationships. All this is probably reflected in the finding that students considered themselves not to be doing well enough in Social support, Life-appreciation and Stress-management.

The food consumption patterns of university students are of particular concern because they also tend to skip meals frequently, eating "fast" foods and snacks. This may be understood because students eat in the school canteen where the service time is short and fixed, and food variety is limited in mainland China. For instance, fresh fruits are seldom available in the university canteen under investigation. As revealed by the survey of adolescent health risk behaviors in a Chinese city in 2005, the highest incidence of skipping breakfast occurred among the university student group [45].

In addition, this study provides evidence of gender and grade differences in the choice of health-promoting lifestyles. Female students were more likely to take a healthy diet but males engaged in more physical exercise, which is consistent with the findings of the Hong Kong Federation of Youth Groups poll [46]. Female students showed more confidence than male students in the Social support dimension (mean score 72.16 versus 63.54) and were far more capable than male students in the use of interpersonal relationships to maintain their psychosocial well-being.

Nutrition behavior was better among senior than junior students, probably because according to the curriculum, nutrition courses are provided to senior students. In terms of life-appreciation, this study revealed that junior students were far more capable than senior students, which may be because the senior students had less enthusiasm for university life owing to a longer time of sensitization.

Although personal characteristics such as gender affect health behaviors, they are seldom incorporated into health interventions since personal characteristics cannot be changed. Health education programs should be planned to stimulate the interests of different students according to their inclinations and characteristics.

There are at least four limitations in the present study. First, no detailed information about non-responders was collected. However, since the response rate was high (84.2%), the bias due to missing information on non-respondents, if any, should be small. Secondly, although the interviewers received uniform training, the interviewers' explanation might still have influenced the results, and this was difficult to evaluate. Thirdly, the subjects in this study were older adolescents with a mean age of 21 and a range of 16-25; the ages 15-25 are usually considered adolescent in mainland China. This should particularly be noted when our results are compared with findings from other regions such as the US, where 'adolescent' is defined as those under the age of 21. Lastly but most importantly, all the subjects were picked from only one university, so the results may provide useful information about only those students' health behaviors. It is not prudent to generalize the results to the whole population of university students or adolescents in mainland China. Students in a medical university are more likely to adopt a healthy lifestyle because of the influence of the medical environment, although this study showed no differences in any dimensions of AHP between medicine specialties and non-medicine specialties. Therefore, in this sense, a large-scale investigation at non-medicine universities should be launched for further study.

## Conclusion

The AHP scale is a valid and reliable instrument for evaluating health-promoting lifestyles of adolescents in mainland China. The score of health-promoting styles in a university in Guangzhou was quite low. The findings suggest that health education in regard to nutrition, exercise, and health responsibility should be strengthened by the university authority, and student affairs administrators should provide facilities to meet the demands of choosing health-promoting behaviors. However, a further large-scale investigation needs to be conducted in multiple regions in mainland China in order to evaluate adolescent health-promoting behaviors and associated factors more fully before the findings are applied widely to the establishment of a health-promoting intervention.

## Competing interests

The authors declare that they have no competing interests.

## Authors' contributions

DW conceived of the study, assisted with the survey, completed the statistical analyses and drafted the manuscript. CQO assisted with data analysis and paper drafting and finalized the manuscript. MYC participated in the design and coordination of the study. ND participated in literature retrieval. All authors read and approved the final manuscript.

## Pre-publication history

The pre-publication history for this paper can be accessed here:


